# Chemokine-Independent VLA-4/VCAM-1-Mediated Rolling and Arrest of B16 Melanoma Cells Under Shear

**DOI:** 10.3390/ijms27083649

**Published:** 2026-04-19

**Authors:** Robert H. Eibl

**Affiliations:** 1 I.L. Weissman Laboratory, Department of Pathology, Stanford University, Stanford, CA 94305, USA; robert.eibl@alumni.dkfz.de; 2 E.C. Butcher Laboratory, Department of Pathology, Stanford University, Stanford, CA 94305, USA; 3 Center for Molecular Biology and Medicine, Veterans Affairs Health Care System, Palo Alto, CA 94304, USA

**Keywords:** VLA-4, VCAM-1, melanoma metastasis, tumor cell adhesion, rolling adhesion, shear flow, flow chamber, endothelial adhesion, integrins

## Abstract

Integrins and other cell adhesion molecules play a critical role in the migration and homing of leukocytes. This study investigates whether metastatic tumor cells can exploit leukocyte-like rolling and arrest mechanisms during early vascular steps of metastatic dissemination. B16 melanoma cell adhesion to activated bEnd.3 endothelial monolayers or immobilized VCAM-1 were analyzed under defined shear flow using a parallel-plate chamber. Function-blocking antibodies, divalent cation modulation, pertussis toxin, and low-temperature conditions were used as classical controls. B16-BL6 melanoma cells exhibited robust VLA-4-dependent rolling and arrest on activated endothelial monolayers and on immobilized VCAM-1 under physiological shear stresses (0.7–2 dyn/cm^2^), independent of chemokine-related Gαi signaling. These findings identify a chemokine-independent mechanism of VLA-4-mediated vascular capture by melanoma cells under shear flow, providing a potential mechanistic basis for early steps in metastatic dissemination.

## 1. Introduction

Major steps in the metastatic cascade can be outlined as follows: detachment of cancer cells from the primary tumor; invasion of the extracellular matrix; intravasation; arrest in the blood vessels at secondary sites; extravasation; and colonization and proliferation [1]. These steps appear to be similar to the physiologic steps involved in leukocyte migration and homing, and similar cell adhesion receptors could contribute to both processes. This study compares the multistep paradigm of leukocyte homing [2,3] with similar steps involved in the metastasis of non-hematopoietic cancer cells. Two major adhesive steps have become a hallmark for circulating leukocytes: rolling and integrin-mediated arrest [4].

Other adhesion molecules have been described to support at least one of these steps, including CD44 [5] (Hubbe and Eibl: submitted). Interestingly, CD44 has been correlated to metastasis formation or tumor progression in some tumors [6,7], but the mechanism remains to be elucidated.

In addition, chemokines have been found to trigger the activation of integrins and rapid arrest of subsets of hematopoietic cells [8]. VLA-4 has been identified to mediate initial tethering, rolling and arrest of leukocytes to its endothelial ligand vascular cell adhesion molecule-1 (VCAM-1) [2,9], whereas α4β7 integrin can mediate rolling and arrest on mucosal addressin cell adhesion molecule 1 (MadCAM-1) for leukocytes homing to the Peyer’s patches in the gut.

The B16-BL6 subline represents a highly invasive and metastatic variant of B16 melanoma, originally developed by in vivo selection for enhanced tissue invasion, and is widely used to study early steps of tumor dissemination. Interestingly, in B16-BL6 melanoma, VLA-4 is highly expressed and has been found to contribute to homotypic aggregation, which binds the cells together and inhibits the early event of metastasis, when cells leave a tumor [10]. This suggests a paradoxical role of VLA-4 in different stages of metastatic progression. Classical models emphasized passive mechanical trapping of tumor cells [11], while the organ-specific distribution of metastases is consistent with the “seed and soil” hypothesis originally proposed by Paget [12]. Early evidence that endothelial adhesion molecules can actively influence metastatic patterns was provided by transgenic mouse models expressing E-selectin, in which expression of appropriate selectin ligands on B16 melanoma cells redirected metastatic colonization from lung to liver [13]. These findings suggested that vascular adhesion receptors can function as active determinants of metastatic organ tropism rather than passive contributors to tumor cell entrapment. In addition, previous studies have implicated VLA-4/VCAM-1 interactions in melanoma cell adhesion to vascular endothelium and in metastatic dissemination [14,15,16,17].

**Figure 1:**
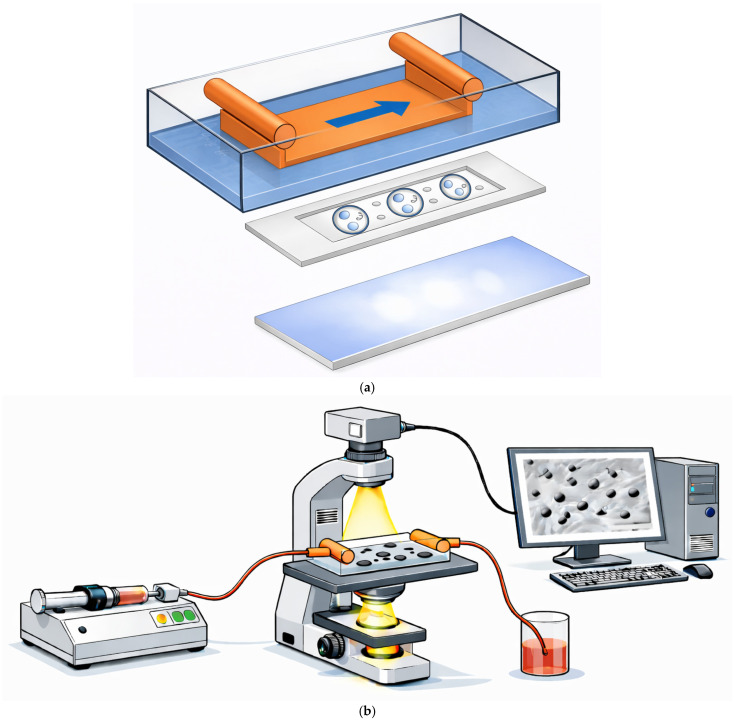
Parallel-plate flow chamber assay. (**a**) Schematic of the custom-made transparent flow chamber (gap height 250 µm; not drawn to scale). The arrow indicates the direction of flow. (**b**) Tumor cells are perfused through the chamber using a syringe pump and analyzed under a defined laminar shear stress. Rolling and arrest are visualized on TNF-α–activated bEnd.3 endothelial monolayers or on slides coated with immobilized adhesion molecules (e.g., VCAM-1) using an inverted microscope equipped with a CCD camera and video acquisition.

Based on these observations, the present study examined whether metastatic tumor cells can exploit leukocyte-like rolling and arrest mechanisms under defined shear conditions using a parallel-plate flow chamber system (Figure 1) and whether VLA-4/VCAM-1 contributes to this process in B16 melanoma cells.

## 2. Results

To explore whether metastatic tumor cells can engage leukocyte-like adhesion behaviors under shear, we used a parallel-plate flow chamber assay as a flow-based analysis system to assess rolling and arrest interactions on activated endothelial monolayers. A panel of murine and human tumor cell lines was examined under identical flow conditions, and adhesive behavior was evaluated using a semi-quantitative scoring approach as described in Section 4.7.

### 2.1. Rolling and Arrest of Metastatic Tumor Cells Under Flow Conditions on Activated Endothelial Monolayers

Using the flow chamber assay, multiple tumor cell lines were systematically examined for their ability to undergo rolling and arrest under physiological shear stress. As summarized in Table 1, most tested tumor cell lines showed no detectable rolling or arrest under these conditions.

**Figure 2:**
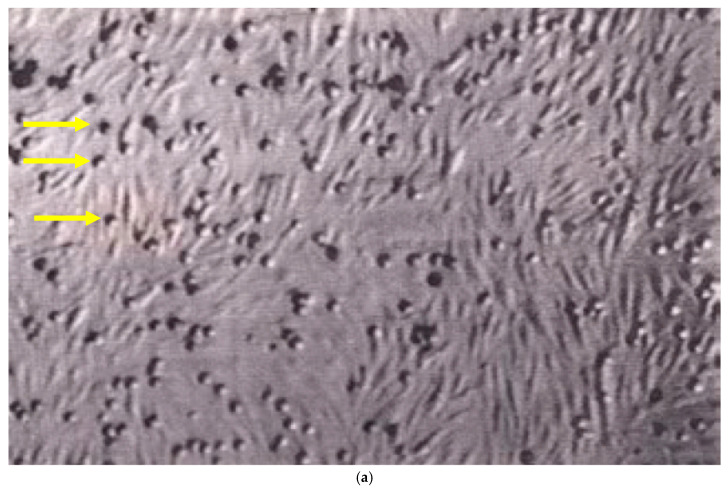

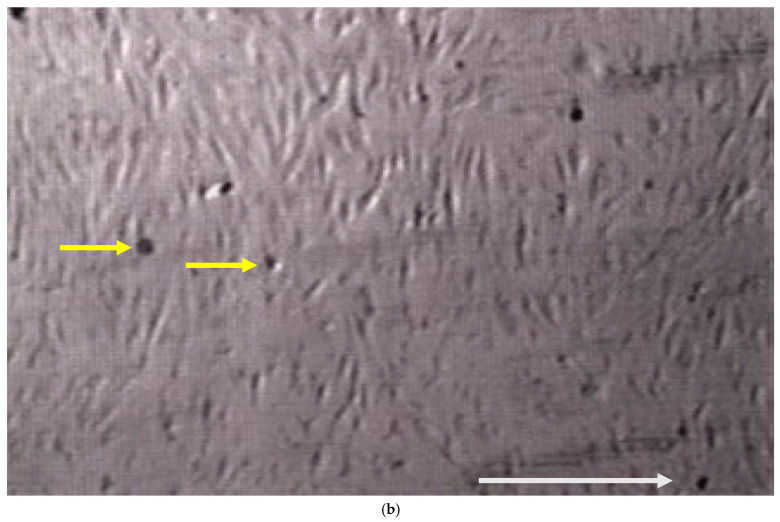
VLA-4-dependent rolling and arrest of B16 melanoma cells on activated endothelium under flow. (**a**) Representative video frame showing single B16-BL6 melanoma cells interacting with TNF-α–activated bEnd.3 monolayers under laminar flow (0.7 dyn/cm^2^), displaying rolling with frequent rapid arrest. (**b**) Pretreatment of B16 cells with a function-blocking anti-VLA-4-antibody (PS/2) strongly reduces rolling/arrest interactions on activated bEnd.3. Yellow arrows indicate representative round interacting cells, while faint elongated streaks represent fast-moving cells in the direction of flow (white arrow).

In contrast, B16-BL6 melanoma cells consistently displayed rolling interactions that frequently transitioned to arrest at shear stresses between 0.7 and 2 dyn/cm^2^ (Figure 2a). A small number of other tumor cell lines exhibited limited rolling behavior; however, these interactions were infrequent and did not progress to stable arrest. These observations identified B16-BL6 melanoma cells as a candidate cell line for further mechanistic analysis of rolling-to-arrest transitions under flow.

### 2.2. Blocking VLA-4 or VCAM-1 Abolishes Rolling and Arrest of B16 Melanoma Cells

Based on the screening results, B16-BL6 melanoma cells were selected for targeted blocking experiments to assess the molecular basis of the observed rolling and arrest behavior. Pretreatment of B16-BL6 cells with a function-blocking anti-α4 integrin antibody (PS/2), or blockade of endothelial VCAM-1 with MK2.7, markedly reduced rolling and arrest interactions under flow (Figure 2b).

In contrast, control antibodies directed against CD44 or MadCAM-1 had no detectable effect on adhesive behavior. These findings are consistent with a dominant contribution of the VLA-4/VCAM-1 interaction to the rolling and arrest phenotype observed for B16-BL6 melanoma cells under the screening conditions used.

### 2.3. Immobilized VCAM-1 Supports Rolling Sufficiency

**Figure 3:**
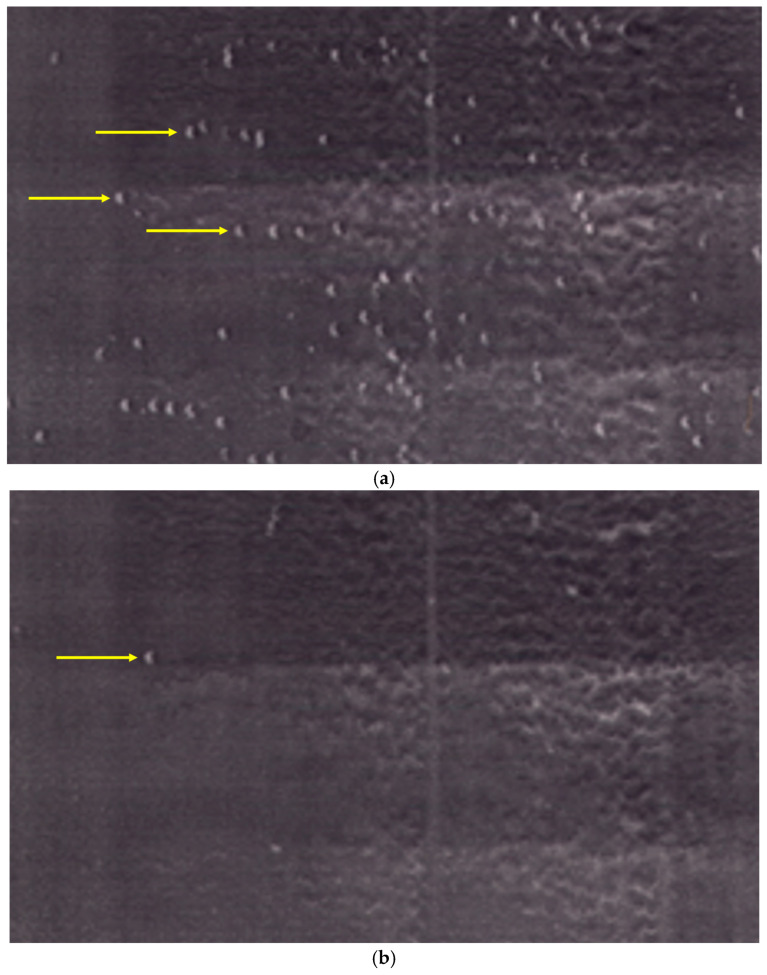
Rolling of B16 melanoma cells on immobilized VCAM-1 under flow. (**a**) B16-BL6 melanoma cells roll on slides coated with purified VCAM-1 under laminar flow (0.7–2 dyn/cm^2^). (**b**) Anti-VLA-4-antibody (PS/2) markedly inhibits rolling on immobilized VCAM-1. Note: Only B16 cells are present in this assay (yellow arrows); cells appear round and may show a small specular reflection.

To determine whether VCAM-1 alone was sufficient to support the rolling behavior observed in the screening assay, B16-BL6 melanoma cells were perfused over surfaces coated with purified VCAM-1. Under these conditions, B16-BL6 cells exhibited rolling interactions with limited firm arrest at comparable shear stresses (Figure 3a).

Rolling interactions on immobilized VCAM-1 were strongly reduced by anti-α4 integrin treatment or by antibody blockade of VCAM-1 (Figure 3b), whereas control antibodies had no detectable effect. These results indicate that the VLA-4/VCAM-1 pair is sufficient to support rolling interactions in this system and that additional endothelial factors may contribute to the rapid arrest observed on activated monolayers.

### 2.4. Further Controls: No Effect of Chemokine, PTX, or Mg^2+^

Additional control conditions were examined to assess whether chemokine-dependent signaling pathways contributed detectably to the observed adhesion behavior. These conditions and their expected versus observed effects are summarized in Table 2.

**Figure 4:**
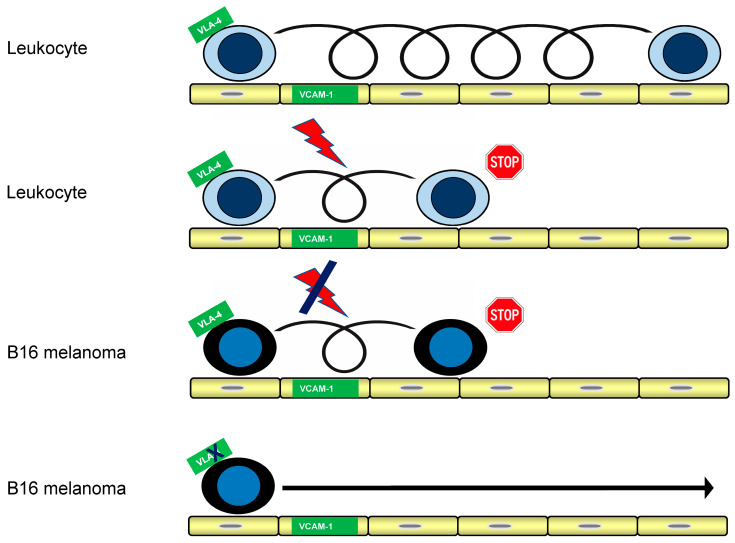
Distinct adhesion mechanisms governing leukocyte versus B16 melanoma rolling-to-arrest transitions. Schematic comparison of leukocyte versus B16 melanoma interactions with VCAM-1 under laminar shear. In leukocytes, rolling is followed by chemokine-dependent Gαi -mediated signaling, which promotes integrin-dependent arrest (PTX-sensitive). In contrast, B16-BL6 melanoma cells roll and undergo rapid arrest in a chemokine-independent, PTX-insensitive manner. Both rolling and arrest are inhibited by VLA-4 blockade. Symbols indicate the presence (lightning) or absence (crossed lightning) of chemokine-dependent signaling. The arrow indicates the direction of flow. The schematic summarizes the experimental findings and is not drawn to scale.

Within the qualitative resolution of the screening assay, treatment with CXCL12, pertussis toxin, or Mg^2+^ supplementation did not produce observable changes in rolling or arrest behavior of B16-BL6 cells. In contrast, chelation of divalent cations by EDTA or incubation at 4 °C abolished detectable adhesive interactions. Together, these findings suggest that, under the conditions examined, VLA-4-mediated rolling and arrest of B16-BL6 melanoma cells can occur independently of classical chemokine-triggered signaling through Gαi proteins, as summarized schematically in Figure 4 (Table 2). Together, these findings are consistent with established models of leukocyte integrin activation but indicate that B16 melanoma cells bypass these canonical regulatory pathways under the conditions tested.

### 2.5. CD44-HA Axis

Although B16-BL6 melanoma cells displayed surface expression of CD44, no detectable CD44-dependent rolling interactions were observed under any of the screening conditions tested. Because these experiments addressed a distinct candidate adhesion pathway, they are described separately from the VLA-4/chemokine-related control conditions summarized in Table 2. Pretreatment with hyaluronidase, reduction in VLA-4-mediated interactions, or experiments performed at low temperature did not reveal alternative CD44-dependent adhesion pathways.

In contrast, BW5147 T-lymphoma cells served as a known positive control and exhibited robust CD44-dependent rolling under identical conditions. These results indicate that CD44 does not measurably contribute to rolling or arrest of B16-BL6 melanoma cells within the sensitivity of the present screening assay.

## 3. Discussion

The role of leukocyte-like adhesion mechanisms in hematogenous metastasis has long been debated. Classical models emphasized passive mechanical trapping of large tumor cells within small capillary beds as a dominant determinant of metastatic arrest [11]. However, accumulating evidence indicates that active, receptor-mediated interactions between tumor cells and the endothelium can decisively influence metastatic efficiency and organ targeting. Experimental flow-based studies have further demonstrated that melanoma and other tumor cells can adhere to endothelial monolayers under defined shear conditions, supporting the concept that circulating tumor cells can actively engage the vascular wall rather than being retained solely by mechanical trapping. In a seminal study, Stamenkovic and colleagues demonstrated that enforced expression of E-selectin in vivo was sufficient to redirect the metastatic pattern of B16 melanoma cells expressing appropriate ligands, providing direct experimental evidence that endothelial adhesion molecules can actively instruct tumor cell localization [13].

Building on this conceptual framework, the present study employed a parallel-plate flow chamber assay as an exploratory screening approach to examine whether metastatic tumor cells can engage leukocyte-like rolling and arrest behaviors under physiological shear conditions. Although the present experiments were performed in an in vitro flow system, the parallel-plate flow chamber reproduces defined physiological shear conditions that closely mimic hemodynamic forces in post-capillary venules [18], where leukocyte adhesion and tumor cell arrest are known to occur.

Screening across a diverse panel of tumor cell lines revealed that such interactions were not broadly distributed but rather restricted to a limited subset of cells. Within this context, B16-BL6 melanoma cells emerged as a candidate model displaying consistent rolling and frequent transition to arrest on activated endothelial monolayers. To our knowledge, leukocyte-like rolling-to-arrest behavior mediated by VLA-4/VCAM-1 has not previously been demonstrated for non-hematopoietic tumor cells under defined shear conditions.

A limitation of this study is its qualitative, screening-based design; however, this approach was intentionally chosen to identify rare but biologically meaningful adhesion phenotypes under physiological shear conditions. Guided by these screening observations, subsequent experiments focused on defining the molecular basis of this phenotype.

Blocking studies identified the VLA-4/VCAM-1 interaction as the dominant adhesion pathway contributing to both rolling and arrest of B16-BL6 melanoma cells under flow. In leukocytes, VLA-4 is well established to support not only firm adhesion but also, in varying amounts, rolling interactions on VCAM-1, depending on activation state and cellular context [2,9,19]. The rolling behavior observed here is therefore consistent with known VLA-4 function. The striking observation in the present system is not the involvement of VLA-4 in rolling per se, but the rapid transition to firm arrest in the absence of detectable chemokine/Gαi signaling. Together, these findings support a distinct adhesion behavior in which tumor cells combine leukocyte-like rolling with rapid arrest in the absence of detectable chemokine/Gαi signaling. Importantly, this behavior links leukocyte adhesion principles with tumor cell vascular interactions and highlights a mechanism that is not typically considered in current models of metastatic arrest.

Previous work has also implicated VLA-4/VCAM-1 interactions in melanoma cell adhesion to the vascular endothelium and in promoting transendothelial migration during metastatic dissemination. However, these studies did not address the dynamic rolling-to-arrest transition under physiological shear conditions examined here [14]. However, differences between immobilized ligand and endothelial presentation may also reflect variations in ligand density or spatial organization, which were not specifically controlled for in the present study. These findings indicate that metastatic melanoma cells can engage integrin-mediated vascular capture directly under shear conditions, without evidence for a requirement of a chemokine-dependent activation step as typically observed for leukocyte arrest. This conceptual distinction is summarized schematically in Figure 4 and highlights a chemokine-independent pathway of integrin-mediated vascular capture.

B16-BL6 represents a highly invasive and metastatic subline of B16 melanoma, originally derived by in vivo selection for enhanced metastatic capacity (B16-F10) and subsequently selected for increased tissue invasiveness (B16-F10BL6). This subline has been widely used to study the early steps of tumor dissemination [20]. While the present experiments were conducted in a murine tumor–endothelial system, the VLA-4/VCAM-1 interaction is highly conserved across species, supporting the broader relevance of these findings beyond the murine context.

In leukocytes, rapid arrest under flow usually requires chemokine-triggered integrin activation, typically mediated by chemokine receptor signaling through Gαi proteins. This signaling pathway can be effectively blocked by pertussis toxin (PTX) [8]. In contrast, B16-BL6 melanoma cells were completely unresponsive to PTX, to chemokine CXCL12 stimulation alone, or to combined chemokine/PTX treatment. None of these conditions altered VLA-4-dependent rolling or arrest. These findings indicate that VLA-4-mediated adhesion in B16 melanoma cells operates independently of canonical chemokine-induced Gαi signaling. Notably, chemokine-independent α4-integrin function has also been observed in leukocyte systems in vivo. Vajkoczy et al. demonstrated in an EAE mouse model that encephalitogenic T cell blasts are captured on CNS microvessels via α4-integrin/VCAM-1 interactions in a G protein–independent manner [21]. These findings provide a rare in vivo precedent for chemokine-independent vascular capture and support the biological plausibility of the mechanism observed here in tumor cells under defined shear conditions. However, these studies did not address tumor cells or the dynamic transition from rolling to arrest under defined shear conditions. The chemokine-independent functionality of VLA-4 may reflect differences consistent with altered integrin regulation at the level of receptor avidity or nanoscale organization rather than classical affinity modulation. VLA-4 on B16-BL6 supports rolling and rapid arrest without detectable chemokine/Gαi dependence under the conditions tested. Atomic force microscopy (AFM)–based force spectroscopy has subsequently been employed by the author to probe such interactions at the single-receptor level between living cells (B16–bEnd.3) [22,23,24], or between B16 cells and immobilized VCAM-1, with comparative measurements performed using lymphoma cells under chemokine CXCL12 stimulation (Eibl, R.H., unpublished observations). These independent measurements are fully consistent with the specificity and the chemokine-independent adhesion behavior observed in the present study.

Despite high surface expression of CD44 on B16-BL6 cells, no binding to hyaluronan and no CD44-dependent rolling were detectable. This contrasts with leukocyte and lymphoma control cells, where CD44–hyaluronan interactions readily support rolling under similar conditions. In several non-B16 tumor cell lines, limited and transient rolling was observed, consistent with selectin-dependent mechanisms previously described for mucin-rich carcinoma cells. Previous studies demonstrated selectin-dependent rolling of carcinoma cells under flow conditions, mediated, for example, by CD24–P-selectin interactions [25,26]. However, such rolling rarely progressed to stable arrest and was fundamentally distinct from the efficient rolling-to-arrest transition mediated by VLA-4/VCAM-1 in B16-BL6 melanoma cells. These observations suggest that tumor cell rolling is mechanistically heterogeneous and that integrin-mediated rolling-to-arrest transitions may represent a specialized adhesion strategy of selected metastatic tumor cells.

Together, these findings argue against a purely mechanical model of metastatic arrest and support a framework in which receptor-mediated adhesion mechanisms operate in a selective and context-dependent manner. While physical constraints and vascular geometry may contribute to initial tumor cell retention, the present screening-to-mechanism analysis indicates that only specific tumor subpopulations possess the molecular repertoire required for efficient rolling-to-arrest transitions under flow. This concept is consistent with the metastatic heterogeneity framework proposed by Fidler, in which only specific tumor cell subpopulations possess the properties required for successful metastatic colonization [27]. In this context, VLA-4/VCAM-1-mediated adhesion reconciles previously paradoxical observations in B16-BL6 melanoma, whereby α4 integrin–dependent homotypic aggregation can suppress dissemination from primary tumors, yet enable efficient endothelial docking once tumor cells enter the circulation. These results underscore the importance of distinguishing passive entrapment from active vascular engagement when interpreting early metastatic events and suggest that endothelial adhesion pathways may represent underappreciated, stage-specific modulators of metastatic seeding.

Parallel-plate flow chamber systems reproduce key biophysical parameters of the vascular microenvironment, including defined shear forces and endothelial presentation of adhesion molecules, and are widely used to model early vascular docking events during leukocyte trafficking and tumor cell dissemination.

The restriction of this phenotype largely to B16 melanoma cells in the present screen further supports the conclusion that efficient rolling-to-arrest transitions under shear depend on specific adhesion receptor repertoires rather than representing a general property of tumor cells.

## 4. Materials and Methods

### 4.1. Cells

Murine B16-BL6 melanoma cells (also referred to as B16-F10BL6) were provided and recommended for this project by Dr. I.J. Fidler. The B16-BL6 variant was selected in vivo for enhanced metastatic and invasive properties from B16-F10 (www.atcc.org, ATCC CRL-6475). Throughout this study, the term B16 refers exclusively to this subline [20]. Cells were grown in DMEM, containing 10% fetal calf serum and antibiotics. Cells were harvested at approximately 70% confluence following a short exposure to 0.5 mM EDTA. Single-cell suspensions (1–5 × 10^5^ cells/mL) were used in laminar flow assays.

bEnd.3 cells, (gift from M. Hubbe and E.C. Butcher, ATCC CRL-2299), a mouse brain endothelial cell line derived from primary brain endothelial cells transformed with polyomavirus middle T antigen [28], was maintained in complete DMEM (DMEM supplemented with 5% fetal bovine serum [endotoxin < 10 pg/mL; Gemini Scientific, West Sacramento, CA, USA] and 5% fetal Clone [Hyclone Laboratories, Logan, UT, USA]).

Selected other cell lines were used to setup the flow system for visual screening of rolling and arrest and were obtained from ATCC: OVCAR-3 (ATCC HTB-161), COLO205 (ATCC CCL-222), MDA-MB-231 (ATCC HTB-26), MCF7 (ATCC HTB-22), BW5147 (ATCC TIB-47), HUVEC (ATCC PCS-100-010), LS174T (ATCC CL-188), HT-29 (ATCC HTB-38), Caco-2 (ATCC HTB-37), and as a gift from V. Sung and D. Feldman: LNCaP (ATCC CRL-1740), PC-3 (ATCC CRL-1435), DU145 (ATCC HTB-81).

### 4.2. Antibodies and Reagents

PS/2 (rat IgG2b, anti-ms α4 integrin [29]): blocking the α4 integrin chain (CD49d) inhibited adhesion to VCAM-1, consistent with α4β1 (VLA-4) as the functional integrin in this assay; MK2.7 (anti-VCAM-1); control antibodies: MECA-367 (anti-MadCAM-1); anti-b7-integrin; HERMES-3 (ms IgG2a, anti-hu CD44 [30]); 84H10 (anti-hu ICAM-1); L133 (anti-hu CD31); TY1138 (anti-hu VCAM-1); WAPS1.2 (anti-hu P-selectin [31]); DREG56 (ms IgG1, anti-hu L-selectin [31]); 9B5 (anti-hu CD44); MJ64 and IM7.8.1 (anti-ms CD44); Mel-14 (anti-ms L-selectin [32]); MI/70 (anti-ms αM [33]); TIB213 (anti-ms αL); 30G12 (rat IgG2a, anti-ms CD45 [34]); all antibodies were provided by E.C. Butcher. TNF-α was a gift from R. Ettinger and H.O. McDevitt. Mg^2+^; chemokine CXCL12/SDF-1 and pertussis toxin (gift from J.J. Campbell and E.C. Butcher).

### 4.3. Purified Proteins

Native mouse VCAM-1 and ICAM-1 proteins were provided by E.C. Butcher and were purified as described earlier [35] by using MAbs MK2.7 and YN1/1.7.4 [19].

### 4.4. Endothelial Monolayer Activation

Confluent monolayers of bEnd.3 cells were grown on microscopic slides (Superfrost Microscope Slides, Erie Scientific, Portsmouth, NH, USA) and activated with TNF-α (1 ng/mL; R&D Systems, Minneapolis, MN, USA) 18 h prior to the flow assay. Preliminary experiments also used IL-1β and LPS, respectively.

### 4.5. Cell Preparation

All cells were cultured under standard cell culture conditions (37 °C, FCS, antibiotics). Cells were normally split 1–3 days before use. On the day of the experiment, single-cell suspensions were prepared using enzyme-free Cell suspension buffer (Gibco, Thermo Fisher Scientific, Waltham, MA, USA) and, if needed, a standard cell scraper. After washing and resuspending steps, including short equilibration in a 37 °C water bath (except for experiments in the cold room), cells were used immediately.

### 4.6. Flow Chamber Assay

The custom-made, transparent parallel-plate flow chamber with a gap of 250 µm was designed as described elsewhere [36]. A syringe pump (Harvard Apparatus, Inc, Holliston, MA, USA) generated a continuous flow. Flow rate and chamber geometry defined the laminar shear stress [18,37], typically chosen between 0.7 and 2 dyn/cm^2^, but also up to 10 dyn/cm^2^. Briefly, glass slides (Superfrost, Erie Scientific, Portsmouth, NH, USA) with confluent endothelial monolayers or immobilized protein (VCAM-1, ICAM-1) were assembled in the flow chamber and mounted to an inverted phase-contrast microscope (Nikon, Tokyo, Japan) with a 20× objective. A video setup, including a camera, VCR, monitor and a timer signal, was used to record the adhesive interactions for later analysis. Video recordings (30 fps) typically covered several minutes per condition. Semi-quantitative scoring was based on consistent visual assessment across independent experiments rather than frame-by-frame tracking, as the aim of the assay was to detect robust and reproducible adhesion phenotypes rather than to derive kinetic parameters. Cell concentrations were kept within a defined range to ensure sufficient observation frequency without altering qualitative interaction patterns.

### 4.7. Quantification and Statistical Analysis

Independent experiments were performed in triplicate. The parallel-plate flow chamber assay was used as a systematic flow-based analysis to identify cell lines capable of rolling and/or arrest under defined laminar shear conditions. Adhesive interactions were evaluated using a semi-quantitative scoring system based on repeated analysis of recorded flow chamber videos under identical shear conditions. Rolling and arrest behavior was classified using a semi-quantitative scale (−, +, ++, +++), as summarized in Table 1. The scale was used to distinguish clearly between absent, weak, and strong adhesion phenotypes. Intermediate categories were not always observed in a given screening dataset.

(−) indicates no detectable rolling or arrest, (+) infrequent or weak interactions, (++) moderate interactions, and (+++) robust and reproducible rolling and/or arrest observed across multiple fields of view. Scoring was consistent between independent experiments. Due to the screening-based and qualitative nature of the assay, no inferential statistical analyses were applied.

This semi-quantitative screening approach was chosen because the primary objective of the assay was the identification of rare but biologically meaningful adhesion phenotypes under physiological shear conditions rather than precise quantification of adhesion frequencies.

## 5. Conclusions

VLA-4 enables chemokine-independent rolling and rapid arrest of B16-BL6 melanoma cells under laminar flow conditions. These findings identify a distinct adhesion mechanism in which melanoma cells employ VLA-4 to mediate both rolling and arrest under shear, independent of chemokine signaling. Distinguishing passive mechanical retention from active, receptor-mediated vascular docking is therefore essential for understanding early metastatic dissemination.

## Figures and Tables

**Table 1:** Overview of tumor cell lines examined under flow conditions on activated bEnd.3 monolayers.

Cell Line	Species	Tumor Type	Rolling Score	Arrest Score	Comment
B16-BL6	Mouse	Melanoma	+++	+++	Rolling and arrest; leukocyte-like behavior
LS174T	Human	Colon carcinoma	+	−	Rolling
COLO205	Human	Colon carcinoma	+	−	Rolling
HT-29	Human	Colon carcinoma	−	−	No rolling or arrest
Caco-2	Human	Colon carcinoma	−	−	No rolling or arrest
OVCAR-3	Human	Ovarian carcinoma	−	−	No rolling or arrest
MDA-MB-231	Human	Breast carcinoma	+	−	Rolling
MCF7	Human	Breast carcinoma	+	−	Rolling
LNCaP	Human	Prostate carcinoma	−	−	No rolling or arrest
PC-3	Human	Prostate carcinoma	−	−	No rolling or arrest
DU145	Human	Prostate carcinoma	−	−	No rolling or arrest
BW5147	Mouse	T-lymphoma	+++	−	CD44-HA dependent rolling; positive control

Semi-quantitative screening of tumor cell rolling and arrest under laminar flow on TNF-α–activated bEnd.3 endothelial monolayers. Rolling and arrest were scored based on visual analysis of recorded flow chamber videos, as described in Section 4.7. Scores indicate the frequency and robustness of adhesive interactions: (−) no detectable rolling or arrest; (+) infrequent or weak interactions; (+++) robust and reproducible rolling and/or arrest observed consistently across independent experiments (*n* = 3). Intermediate categories were not necessarily observed in a given screening dataset.

**Table 2:** Functional control conditions used to assess VLA-4-mediated adhesion under flow, based on established leukocyte adhesion paradigms.

Condition	Established Role in Leukocyte Adhesion (Refs.)	Expected Effect on Adhesion	Observed Effect on B16
Mg^2+^ supplementation	Promotes high-affinity conformation of VLA-4 and other integrins in leukocytes [4,9]	Enhanced rolling and/or arrest	No visible increase in rolling/arrest; no detectable change
Pertussis toxin (PTX)	Blocks Gαi-dependent chemokine receptor signaling [8]	Reduced adhesion, if VLA-4 was chemokine-activated	Rolling/arrest preserved; no detectable change
CXCL12	Activation of VLA-4, if CXCR4 is expressed, rapid arrest [8]	Activation of VLA-4, but not really expected here (control)	Rolling/arrest preserved; no detectable change
CXCL12+PTX	Blocked activation of CXCL12 and any other Gαi-dependent VLA-4 activation pathways [8]	Reduced adhesion, if any chemokines were involved (control, if CXCL12 alone had any effect)	Rolling/arrest preserved; no detectable change
4 °C cold room	Suppresses active signaling and energy-dependent processes	Reduced adhesion	No rolling/adhesion

## Data Availability

The data supporting the findings of this study are available from the corresponding author upon reasonable request.

## Abbreviations

The following abbreviations are used in this manuscript:

AFM	Atomic force microscopy
CD44	Cluster of differentiation 44, hyaluronan receptor
CXCR4	C-X-C chemokine receptor type 4
CXCL12	A chemokine, also known as stromal cell-derived factor 1, SDF-1
DMEM	Dulbecco’s modified Eagle’s medium
EDTA	Ethylenediaminetetraacetic acid
FCS	Fetal calf serum
Gαi	Inhibitory G protein alpha subunit
HA	Hyaluronan
HUVEC	Human umbilical vein endothelial cells
ICAM-1	Intercellular cell adhesion molecule 1
IL-1β	Interleukin-1 beta
LPS	Lipopolysaccharide
MAdCAM-1	Mucosal addressin cell adhesion molecule 1
MAb	Monoclonal antibody
PTX	Pertussis toxin
TNF-α	Tumor necrosis factor alpha
VCAM-1	Vascular cell adhesion molecule 1
VLA-4	Very late antigen, α4β1 integrin
